# Hemorrhagic Meningoencephalomyelitis Due to Ectopic Localization of *Aelurostrongylus abstrusus* in a Cat: First Case Report

**DOI:** 10.3390/ani12020128

**Published:** 2022-01-06

**Authors:** Fernanda Viola Tinoco, Simone Morelli, Marilene de Farias Brito, Gabriela Oliveira Pereira, Mariana Correia Oliveira, Anastasia Diakou, Mariasole Colombo, Antonio Frangipane di Regalbono, Donato Traversa

**Affiliations:** 1 Clinica Veterinária Cães & Gatos, Barra do Piraí 27113-150, Brazil; fvtmedvet@hotmail.com; 2 Faculty of Veterinary Medicine, University of Teramo, 64100 Teramo, Italy; smorelli@unite.it (S.M.); mcolombo@unite.it (M.C.); 3 Department of Epidemiology and Public Health, Federal Rural University of Rio de Janeiro, Seropédica 23897-000, Brazil; mfariasbrito@uol.com.br (M.d.F.B.); gabrielaolivie@gmail.com (G.O.P.); marimedvet2009@gmail.com (M.C.O.); 4 Faculty of Health Sciences, School of Veterinary Medicine, Aristotle University of Thessaloniki, 54124 Thessaloniki, Greece; diakou@vet.auth.gr; 5 Department of Animal Medicine, Production and Health, University of Padova, Legnaro, 35020 Padova, Italy; antonio.frangipane@unipd.it

**Keywords:** parasitic meningoencephalomyelitis, neurological signs, feline aelurostrongylosis, kitten, ectopic localization

## Abstract

**Simple Summary:**

*Aelurostrongylus abstrusus* is the most important parasitic nematode affecting cat airways worldwide. The adult nematodes live in the lungs and cause pneumonia with typical respiratory clinical signs such as cough, nasal and ocular discharge, and difficulties in breathing. The localization of adult *A. abstrusus* in other organs than lungs is not common. This report describes, for the first time, an unusual and fatal localization of *A. abstrusus* within the central nervous system of a kitten displaying neurological signs.

**Abstract:**

The lungworm *Aelurostrongylus abstrusus* is one of the main causes of respiratory diseases in cats worldwide. This report describes the unusual case of a kitten infected with *A. abstrusus* and presented to a veterinary clinic in Brazil with lethargy, dysphagia, non-ambulatory tetraplegia, and pelvic limbs bilateral myoclonus. The clinical picture of the kitten worsened with generalized flaccid tetraplegia and death a few days after hospitalization. At necropsy, hemorrhagic necrosis and subarachnoid hemorrhages were detected in several areas of the central nervous system. Nematode stages were found at post-mortem histological examinations in lungs, cerebellum, subarachnoid space of the brain and spinal cord. Microscopic and molecular (PCRs-coupled-sequencing protocols) examination showed the presence of *A. abstrusus* in histological samples. This study describes the first neurological aelurostrongylosis due to ectopic localization of adult worms in the central nervous system of a cat, causing acute hemorrhagic multifocal meningoencephalomyelitis. Further studies are necessary to elucidate whether unusual localizations and the migration of *A. abstrusus* are more frequent than expected.

## 1. Introduction

Nematodes living in the airways are among the most important causes of respiratory diseases in cats, and, among them, the “cat lungworm” *Aelurostrongylus abstrusus* (Metastrongyloidea, Angiostrongylidae) is spread worldwide [1]. The lifecycle of *A. abstrusus* is indirect. The adults live in the alveoli, alveolar ducts, and bronchioles of domestic and sometimes wild felids [2,3,4]. After mating, females produce eggs that hatch in the airways and release first stage larvae (L1). The L1 reach the pharynx via the muco-ciliary escalator, are swallowed, and then excreted with faeces in the environment [5,6]. Various species of terrestrial gastropods act as intermediate hosts in which L1 develop to the second (L2) and third, infectious, larval stage (L3) [7,8]. Rodents, birds, and reptiles may act as paratenic hosts of *A. abstrusus*. Cats become infected by ingesting infected intermediate or, more frequently, paratenic hosts [4,9].

The clinical severity of feline aelurostrongylosis depends on the age of the cat, immune response, health status, and parasitic burden [1]. In many cases, aelurostrongylosis is mild/subclinical and/or self-limiting, though severe and potentially fatal clinical conditions may occur [1]. The most frequent clinical signs are cough, dyspnea, tachypnea, lethargy, weight loss, and anorexia [1,10]. The diagnosis relies on the identification of L1 in the faeces of infected cats whose identity can be confirmed with molecular methods. Recently, new serological assays have been developed for research purposes [11,12,13].

Though common for angiostrongylid nematodes [14,15,16], aberrant localizations of adult *A. abstrusus* have never been described. The present report describes the clinical implications and pathogenetic features of the first case of a fatal hemorrhagic multifocal meningoencephalomyelitis due to ectopic localization of *A. abstrusus* in the central nervous system (CNS) of a domestic cat.

## 2. Case Details

### 2.1. Clinical Case

A male domestic Brazilian short-haired kitten, approximately 3–4 months old, was adopted from the street in a peri-rural area and presented on the same day to a veterinary clinic in Barra do Piraí, Brazil. The main complaints were lethargy, ataxia, and dysphagia 3 days after adoption.

Clinical examination revealed dehydration, pale mucous membranes, distended abdomen, and wheezing and rhonchi were present at the pulmonary auscultation. Complete blood count showed normocytic, normochromic non-regenerative anemia, and thrombocytopenia. Serum biochemistry revealed hypoproteinemia and hypoalbuminemia. Detailed laboratory alterations are shown in Table 1. Serology for Feline Leukemia Virus (FeLV) was positive, while the animal was negative for Feline Immunodeficiency Virus (FIV) (ELISA SNAP test, IDEXX Laboratories Inc.). The kitten was then hospitalized and underwent supportive therapy with rehydration using Lactated Ringer’s solution (replacement volume of water deficit = 7% of body weight in 24 h).

**Figure 1:**
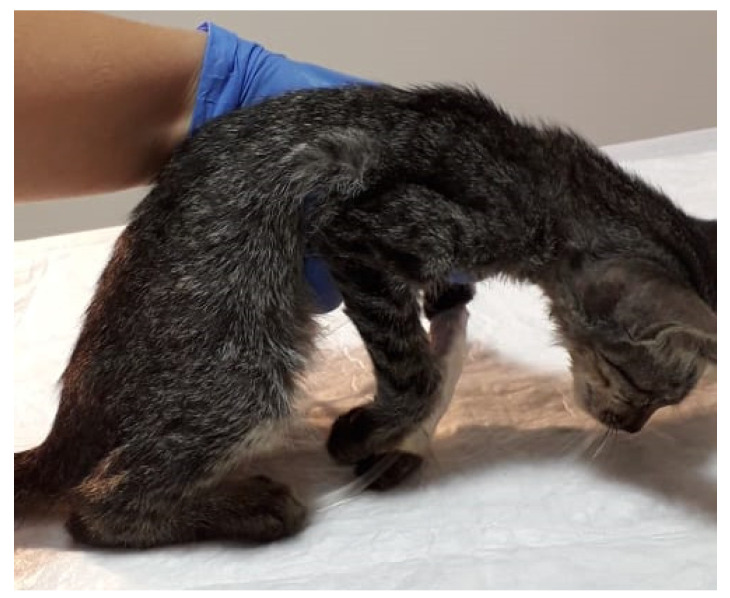
The kitten of the present study showing obtundation and non-ambulatory tetraplegia.

The neurological examination showed non-ambulatory tetraplegia and pelvic limbs bilateral myoclonus. Some superficial reflex was still present at spinal assessment. The day after, the kitten showed severe non-ambulatory tetraplegia (Figure 1), loss of nociception in the pelvic limbs, and trismus. Four days after the hospitalization, the clinical condition of the kitten dramatically worsened with generalized flaccid tetraplegia and the cat died in the clinic 9 days later.

### 2.2. Necropsy and Histopathology

**Figure 2:**
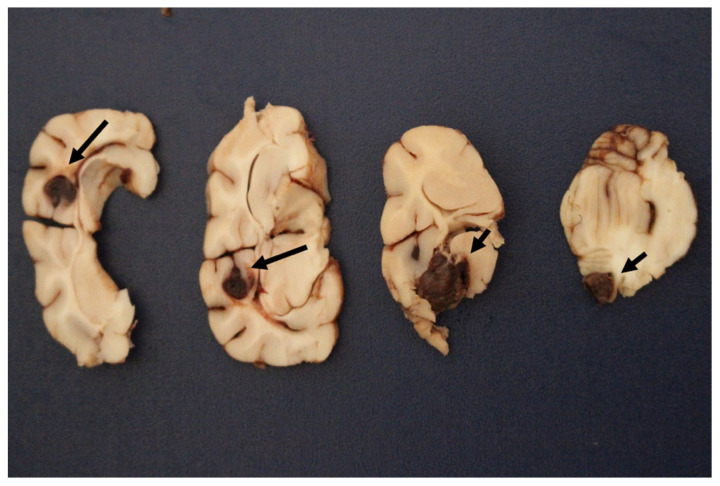
Macroscopic sections of the brain of the kitten. From left to right, hemorrhagic areas were present in the cerebral cortex, corona radiata, diencephalon, and cerebellum (arrows).

**Figure 3:**
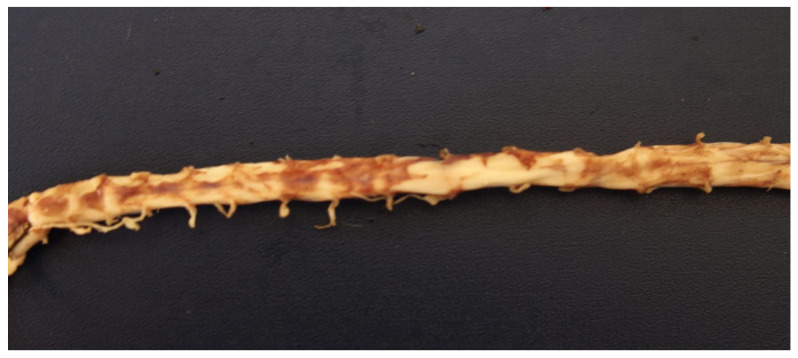
Mild to moderate foci of subarachnoid hemorrhages in the cervical and lumbar tract of the spinal cord of the kitten.

Necropsy was carried out at the Department of Pathological Anatomy of the Federal Rural University of Rio de Janeiro (SAP-UFRRJ) and showed areas of hemorrhagic necrosis in the left frontal telencephalic cortex, cerebellum, and brainstem (Figure 2). Subarachnoid hemorrhages were also present in the cervical and lumbar tract of the spinal cord (Figure 3).

**Figure 4:**
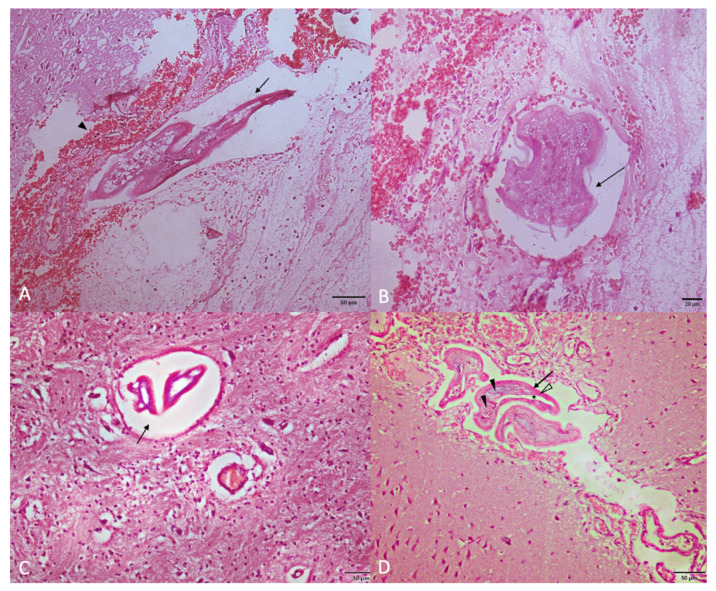
(**A**) Histological section of the brain stained with hematoxylin-eosin. Longitudinal aspect of nematodes (arrow) in the subarachnoid space of the diencephalon, associated with severe hemorrhage (arrowhead). (**B**) Histological section of the brain stained with hematoxylin-eosin. Transverse aspect of a nematode inside a vessel in the subarachnoid space of the diencephalon (arrow). A severe hemorrhage around the vessel is evident. (**C**) Histological section of the brain stained with hematoxylin-eosin. Transverse aspect of nematodes inside of telencephalic brain vessels (arrow). (**D**) Histological section of a vessel in the subarachnoid space of the cerebral cortex, stained with hematoxylin-eosin. Cross sections of nematode with a thin cuticle (arrow), pseudocoelom (asterisk), celomatic musculature (empty arrowhead) and structures representing the internal organs of the parasite (arrowheads).

Nematodes were also detected at the histological examination in the subarachnoid space of the brain and the spinal cord, which showed hemorrhages/congestion and cerebral lymphocytic, eosinophilic, and neutrophilic infiltrates (Figure 4A,B). In the CNS, cross-sections of adult nematodes measuring between 62 and 94 µm in width were found (Figure 4C). Thin cuticle, pseudocoelom, celomatic musculature, and structures representing the internal organs of the parasite were observed in some of the sections (Figure 4D).

**Figure 5:**
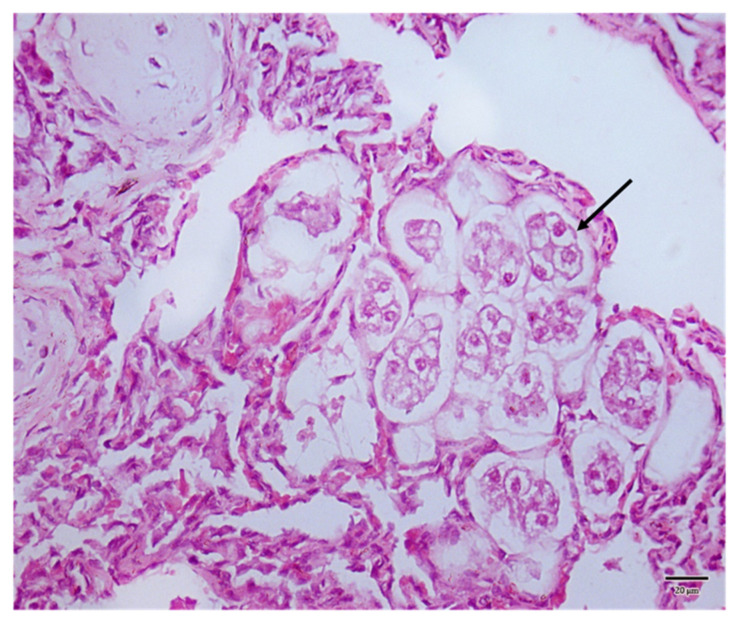
Histological section of the lung stained with hematoxylin-eosin. Parasitic eggs that fill the alveolar lumens are visible (arrow).

At lungs gross examination, nematodes were found in the airways and identified as *A. abstrusus* according to morphological keys [17]. During the histological examination of the lungs, marked atelectasis and severe interstitial inflammatory infiltrate with a predominance of lymphocytes and histiocytes were found. A typical appearance of the lung parenchyma compatible with aelurostrongylosis was observed [18], with the presence of eggs in a 3–8 cell stage in the alveolar lumen (Figure 5).

### 2.3. Molecular Biology

Samples from the lung, cerebellum, and spinal cord were subjected to PCRs-coupled-sequencing protocols specific for four feline Metastrongyloidea nematodes belonging to the Angiostrongylidae (i.e., *Gurltia paralysans*, *Angiostrongylus chabaudi* and *A. abstrusus*) and Crenosomatidae (*Troglostrongylus brevior*) families [12,19]. All samples were positive for *A. abstrusus* at PCR, while no amplicons were generated for the other nematodes. The sequencing showed 100% identity with an *A. abstrusus* isolate from Colombia (Accession Number MH779453).

## 3. Discussion

This study describes the first report of multifocal meningoencephalomyelitis in a cat due to *A. abstrusus*. Measurements of nematodes found in the CNS of the kitten were compatible with adult stages of *A. abstrusus*, whose width ranges between 54 and 100 µm [5,17,20,21]. On the contrary, no parasitic structures compatible with *A. abstrusus* L1, L2, or L3 were found in the CNS in the present case, e.g., their width is 17.6 ± 2.6, 27.6 ± 4.5, and 26.7 ± 1.9 µm, respectively [22].

Such an ectopic localization of *A. abstrusus* adults in the CNS was totally unexpected, as neurological signs due to *A. abstrusus* have never been described before the present case. Thus, other feline parasites were initially suspected as the cause of disease in the referred kitten.

Initially, the clinical picture showed by the kitten of the present study led to the suspicion of infection by *G. paralysans*, as this poorly known angiostrongylid infects the vessels of the subarachnoid space of the spinal cord and parenchyma of felids in South America (including Brazil), and causes life-threatening neurological disorders [23,24].

Other than *G. paralysans,* nematodes of the genus *Angiostrongylus* could have been the cause of the here described neurological picture, as cases of ectopic localizations and aberrant migrations in vertebrates are known for these nematodes [14,15,16]. Adult *Angiostrongylus vasorum* affecting canids have been found in ectopic localizations, e.g., pericardial sac, spleen, kidneys, bladder, eye [14,25]; aberrant larval migrations in different organs and tissues, including the brain and spinal cord, have been described [14,15]. Thus, the closely related species *A. chabaudi* has been considered as a possible cause of the herein reported case, although this parasite has never been recorded thus far in South America. The pathogenic role of *A. chabaudi* for domestic cats is yet to be elucidated [26] and its ability to migrate in the central nervous system, as other species of *Angiostrongylus*, could not be excluded.

*Troglostrongylus brevior* is a crenosomatid parasite of the bronchi of domestic and wild felids. Despite the overlapping biology with that of *Angiostrongylus* spp. and *A. abstrusus*, this lungworm may be transmitted vertically from the queen to the litter [4,27]. This biological feature implies the ability of larvae to establish varying migration patterns throughout the body of a cat. Given that troglostrongylosis is frequent in kittens, *T. brevior* could have been included in the differential diagnosis though never recorded in felids of South America, but only in wild-caught gastropods from Colombia [28].

No evidence of gurltiosis, angiostrongylosis, or troglostrongylosis was found in the kitten at necropsy and microscopic and molecular tests.

*Aelurostrongylus abstrusus* was included in the molecular examinations because of the presence of compatible stages in the lung parenchyma of the kitten. The samples from the lungs and CNS produced PCR amplicons specific for ribosomal sequences of *A. abstrusus*, while none of them tested positive for *G. paralysans*, *A. chabaudi*, or *T. brevior*. This result is surprising as aelurostrongylosis is a typical respiratory disease and the involvement of other organs in the clinical disease has been described only in a single report in a kitten. In this latter case, the cat displayed a life-threatening pneumonia along with enteritis due to massive larval invasion in the intestinal mucosa [29]. Although it was unclear if these were L1 or L3, it was speculated that the phenomenon was due to a hyperinfection syndrome, most likely related to the exposure to a heavily infected intermediate or paratenic host [29]. Another report described the presence of L1 within colon crypts of two cats, with no detailed clinical information available; thus, these might have been incidental findings [30]. In general, it cannot be ruled out that the ingestion of a huge amount of L3 could lead to non-respiratory clinical signs and increase the chances of ectopic localizations. In the present report, an exceptional case of autoinfection could be ruled out as intermediate hosts are necessary for the development of infectious *A. abstrusus* L3. Therefore, it could be hypothesized that an inefficient immune response (e.g., the young age of the animal and/or FeLV positivity) could have triggered an aberrant migration after the ingestion of high amount of infectious L3.

## 4. Conclusions

In conclusion, this report adds new and unexpected knowledge on clinical feline aelurostrongylosis because it represents the first case ever described of an ectopic localization of *A. abstrusus* with a subsequent fatal neurological disease in a naturally infected cat. The kitten was seropositive for FeLV, which is considered to cause neurologic disease in cats [30]. The lack of immunohistochemical identification of the virus in the central nervous system is a limitation of this study as no correlation between the seropositivity and the neurological signs can be safely assumed [31]. However, the presence in the CNS of (i) multifocal hemorrhages, (ii) adult *A. abstrusus*, and (iii) neutrophilic/eosinophilic inflammation of the cerebral parenchyma and in the meninges, demonstrate a CNS aelurostrongylosis. Therefore, ectopic localizations of *A. abstrusus* may occur in kittens, and further studies are encouraged, especially in animals with concomitant diseases that may impair their immune system.

## Figures and Tables

**Table 1:** Complete blood count and blood chemistry alterations of the kitten.

Complete Blood Count	Result	Reference Ranges
Hematocrit%	20.0	25–45
Hemoglobin g/dL	7.1	8.0–17.0
Erythrocytes mil./mm^3^	4.80	5.0–10.0
Reticulocytes% (absolut/mm^3^)	0	0.5–1.0% (27,500–55,000)
Plasma proteins	5.80	6.5–7.5
Platelets/mm^3^	116.000	200,000–800,000
**Serum biochemistry**		
Total protein g/dL	4.0	5.4–7.6
Albumin g/dL	1.7	2.1–3.3
Globulins g/dL	2.3	2.8–5.5

## Data Availability

All study data are presented in the article.
